# Genome sequence of a commensal bacterium, *Enterococcus faecalis* CBA7120, isolated from a Korean fecal sample

**DOI:** 10.1186/s13099-016-0145-x

**Published:** 2016-11-25

**Authors:** Joon Yong Kim, Hye Seon Song, Yeon Bee Kim, Joseph Kwon, Jong-Soon Choi, Yong-Joon Cho, Byung-Yong Kim, Jin-Kyu Rhee, Jinjong Myoung, Young-Do Nam, Seong Woon Roh

**Affiliations:** 1 Biological Disaster Analysis Group, Korea Basic Science Institute, Daejeon, 34133 South Korea; 2 Department of Food Science and Engineering, Ewha Womans University, Seoul, 03760 South Korea; 3 ChunLab Inc., Seoul National University, Seoul, 151-742 South Korea; 4 Korea Zoonosis Research Institute, Chonbuk National University, Jeonju, 561-756 South Korea; 5 Research Group of Gut Microbiome, Korea Food Research Institute, Seongnam, 13539 South Korea; 6 University of Science and Technology, Daejeon, 34113 South Korea

**Keywords:** *Enterococcus faecalis*, Genome sequence, Comparative genomics, Virulence factors

## Abstract

**Background:**

*Enterococcus faecalis*, the type strain of the genus *Enterococcus*, is not only a commensal bacterium in the gastrointestinal tract in vertebrates and invertebrates, but also causes serious disease as an opportunistic pathogen. To date, genome sequences have been published for over four hundred *E. faecalis* strains; however, pathogenicity of these microbes remains complicated. To increase our knowledge of *E. faecalis* virulence factors, we isolated strain CBA7120 from the feces of an 81-year-old female from the Republic of Korea and performed a comparative genomic analysis.

**Results:**

The genome sequence of *E. faecalis* CBA7120 is 3,134,087 bp in length, with a G + C content of 37.35 mol%, and is comprised of four contigs with an N50 value of 2,922,046 bp. The genome showed high similarity with other strains of *E. faecalis*, including OG1RF, T13, 12107 and T20, based on OrthoANI values. Strain CBA7120 contains 374 pan-genome orthologous groups (POGs) as singletons, including “Phages, Prophages, Transposable elements, Plasmids,” “Carbohydrates,” “DNA metabolism,” and “Virulence, Disease and Defense” subsystems. Genes related to multidrug resistance efflux pumps were annotated in the genome.

**Conclusions:**

The comparative genomic analysis of *E. faecalis* strains presented in this study was performed using a variety of analysis methods and will facilitate future identification of hypothetical proteins.

## Background

Genus *Enterococcus* is a common member of the normal intestinal flora in various species, both vertebrates and invertebrates. However, some species in the genus *Enterococcus* are leading causes of highly contagious hospital-acquired infections, including urinary tract, intra-abdominal, pelvic, and soft tissue infections, as well as bacteremia and endocarditis. Thus, members of the genus *Enterococcus* have been extensively studied. The genus *Enterococcus* was first described by Schleifer and Kilpper-Balz [1] and species of the genus *Enterococcus* are Gram-positive, non-spore-forming, facultative anaerobic microbes that produce lactic acid. *Enterococcus faecalis* DSM 20478^T^ is the type species for the genus [2].

The first genome sequence of *Enterococcus* was published by Paulsen in 2003 with *E. faecalis* V583 [2]. To date, more than 400 strains of *E. faecalis* have been sequenced and analyzed. The virulence factors present in *E. faecalis* are well established, and include aggregation substances, surface adhesins, sex pheromones, lipoteichoic acid, extracellular superoxide, the lytic enzymes gelatinase and hyaluronidase, and the toxin cytolysin, but novel virulence factors continue to be reported [3]. In this study, we performed sequencing and genomic analysis of *E. faecalis* CBA7120, isolated from the feces of an 81-year-old female. Comparison of genomic data from *E. faecalis* CBA7120 with other genomes of *E. faecalis* may improve our understanding of the virulence factors and pathogenesis present in *Enterococcus*.

## Methods

### Strain isolation and DNA extraction

*Enterococcus faecalis* CBA7120 was isolated from the feces of an 81-year-old healthy female living in the Republic of Korea and cultivated on modified Eggerth–Gagnon (EG) medium [containing per liter of distilled water: peptone 10 g, Na_2_HPO_4_ 4 g, porcine gastric mucin 2 g, sheep blood 50 ml, agar 15 g] at 37 °C for 24 h in an anaerobic chamber (Coy Laboratory Products). Once a pure culture was obtained, strain CBA7120 was preserved at −80 °C in a suspension of 20% glycerol for long-term storage. Genomic DNA for sequencing was prepared using QuickGene DNA tissue kit S (Kurabo, Japan) and QIAamp DNA extraction Kits (Qiagen, USA).

### Whole genome sequencing, assembly, and gene annotation

A SMRTbell library was constructed according to the Pacific Biosciences protocol “20-kb Template Preparation Using BluePippin Size-selection system (15-kb Size Cutoff)”. The library was sequenced using P6-C4 chemistry on a Pacific Biosciences RS II instrument. The PacBio RS II sequencing system generated 150,292 reads, with an average read length of 8095 bp from one SMRT cell. For the assembly, filtering was performed by Hierarchical Genome Assembly Process (HGAP) version 2 protocol with default parameter. Assembly was performed using the HGAP 2 protocol with default parameters in SMRT Analysis version 2.3 [4]. The assembly was polished with three successive passes through Quiver to reach a final consensus accuracy of >99.988% at 232.798 × coverage. Finally, finished assembly consisted of four contigs. Using RS_Modification_and_Motif_analysis protocol with default parameter in PacBio SMRT analysis pipeline, 4 N6-methyladenine and other six unidentified methylated motifs were identified. Gene prediction was accomplished using Glimmer3 [5] on the Rapid Annotation using Subsystem Technology (RAST; http://rast.nmpdr.org/) server [6], and gene annotation was performed using the SEED and Clusters of Orthologous Groups (COG; http://www.ncbi.nlm.nih.gov/COG/) databases [7, 8]. RNAmmer 1.2 [9] and tRNAscan-SE 1.21 [10] were used to identify rRNA and tRNA sequences, respectively.

### Multilocus sequence typing (MLST)

MLST was performed using the following seven housekeeping genes: glucose-6-phosphate dehydrogenase (*gdh*), glyceraldehyde-3-phosphate dehydrogenase (*gyd*), phosphate ATP binding cassette transporter (*pstS*), glucokinase (*gki*), shikimate-5-dehydrogenase (*aroE*), xanthine phosphoribosyltransferase (*xpt*), and acetyl-CoA acetyltransferase (*yiqL*) using PubMLST (http://pubmlst.org/efaecalis/).

### Comparative genomic analysis

Using data in the NCBI genome database (http://www.ncbi.nlm.nih.gov/genome/), four E. *faecalis* strains were selected as the closest neighbors of strain CBA7120 (>89% symmetric identity): *E*. *faecalis* strain 12107, T13, OG1RF, and T20, and these genomes were used for comparative genomic analysis. The genome of E. *faecalis* TX0031 also showed high symmetric identity, but it was excluded from further analysis because the genome contains a large number of contigs. For whole-genome comparison, the genomes of strain CBA7120 and the other related strains were aligned using the progressive MAUVE algorithm in the MAUVE multiple genome alignment software 2.4.0 [11]. The OrthoANI algorithm was used to assess overall similarity between two genome sequences [12]. OrthoANI values were obtained and a phylogenetic tree was constructed based on OrthoANI analysis of the *E. faecalis* strains CBA7120, 12107, T13, OG1RF, and T20 using the orthologous average nucleotide identity tool [12]. Orthologs in the genomes of strain CBA7120 and other related strains were identified using reciprocal best hit (RBH) in the UBLAST program [13]. Pan-genome orthologous groups (POGs) were constructed using the EzBioCloud Comparative Genomics Database (http://cg.ezbiocloud.net/). For the visualization heat map and the dendrogram based on the gene content (presence or absence) of the genomes of strain CBA7120 and the other four strains, Jaccard coefficients and UPGMA clustering were used to calculate the presence of POGs and genome clustering, respectively. The Venn diagram based on POGs shared between strain CBA7120 and related strains was constructed using the jvenn program [14].

### Quality assurance

**Fig. 1:**
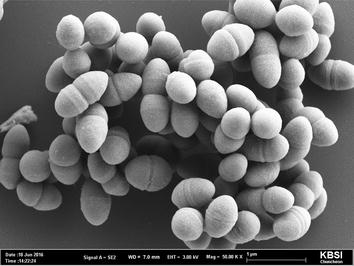
Photomicrograph of *Enterococcus faecalis* CBA7120

To obtain pure cultures, a single colony of strain CBA7120 was repeatedly transferred to fresh modified EG medium more than three times, and confirmed using electron microscopy (Fig. 1). Genomic DNA of strain CBA7120 was purified using an MG Genomic DNA purification kit (Doctor Protein) and the 16S rRNA gene sequence from the draft genome was used to check for contamination.

## Results and discussion

### General features

**Fig. 2:**
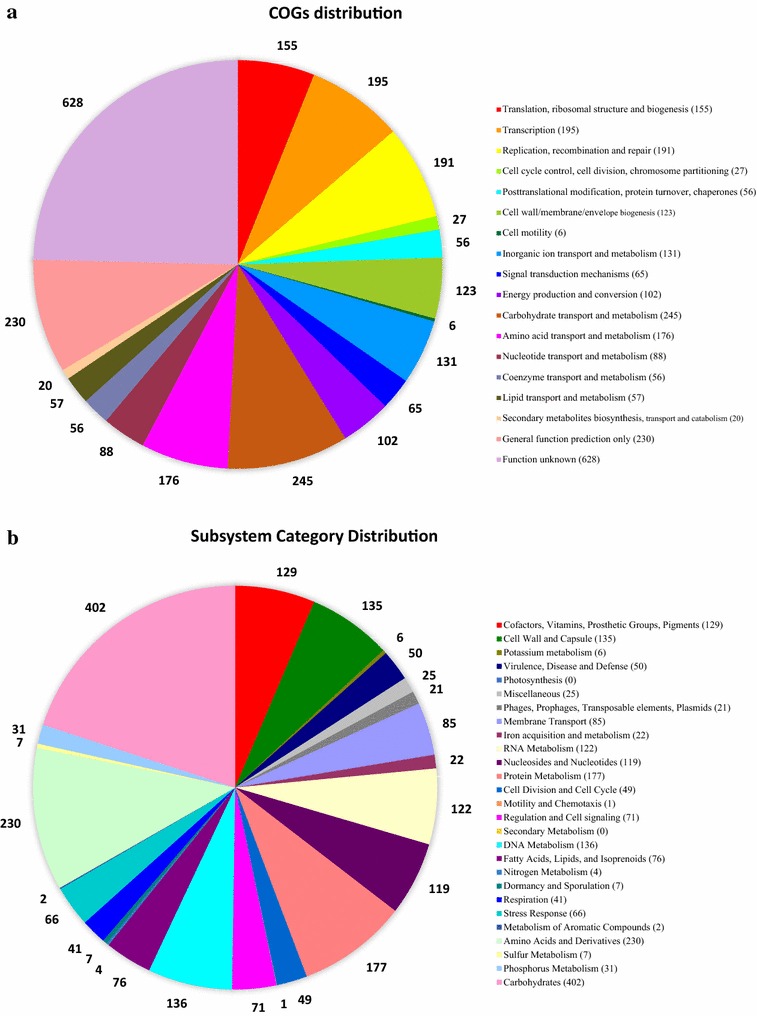
Analysis of annotated genes for *Enterococcus faecalis* CBA7120 based on the SEED (**a**) and COG (**b**) databases

General genome features of *E. faecalis* CBA7120 are shown in Table 1. The genome sequence of strain CBA7120 is 3,134,087 bp in length, with a G + C content of 37.35 mol%, and is comprised of four contigs with an N50 value of 2,922,046 bp. Using BLAST in the NCBI, we found that contig 1, 2 and 4 represented plasmid sequences and contig 3 represented chromosome sequence. The genome contains 3018 coding DNA sequences (CDSs), 60 tRNA genes, and four 16S-23S-5S rRNA operons. The distribution of COGs and SEED subsystems is illustrated in Fig. 2. The most abundant COG categories were G (carbohydrate transport and metabolism), R (general function prediction only), K (transcription), L (replication, recombination and repair), E (amino acid transport and metabolism), J (translation, ribosomal structure and biogenesis), and P (inorganic ion transport and metabolism); the S category (function unknown) was also abundant (Fig. 2a). The SEED subsystems “Carbohydrates”, “Amino Acids and Derivatives”, “Protein Metabolism”, “DNA Metabolism”, “Cell Wall and Capsule”, “Cofactors, Vitamins, Prosthetic Groups, Pigments”, “RNA Metabolism”, and “Nucleosides and Nucleotides” subsystems were most abundant (Fig. 2b).

**Table 1:** General genome features of *Enterococcus faecalis* CBA7120

Item	Values
Finishing quality	Draft
Sequencing platforms	PacBio_20K
Assembler	PacBio SMRT Analysis 2.3.0
Methods reads	150,292
Genome coverage	232.798X
Assembly size (bp)	3,134,087
N50	2,922,046
DNA G + C content (%)	37.35
Total contigs	4
Coding sequences	3018
rRNA genes	4 16S-23S-5S rRNA operon
tRNA genes	60

### Multilocus sequence typing (MLST)

*Enterococcus faecalis* CBA7120 was typed with MLST at loci *gdh* 1, *gyd* 7, *pstS* 9, *gki* 1, *aroE* 1, *xpt* 3, and *yqiL* 1, and was classified as sequence type 5 (ST 5) containing solely strain B343 that was isolated from a chicken product in Spain.

### Comparative genomics of strain CBA7120 with other *E. faecalis* strains

**Fig. 3:**
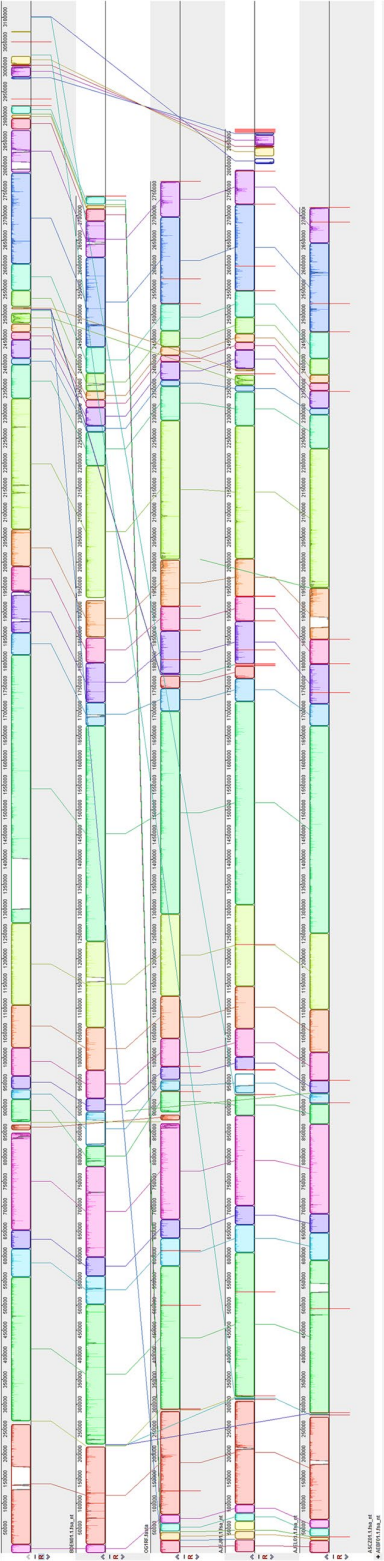
MAUVE alignment of the genome of *Enterococcus faecalis* CBA7120 and the genomes of strains OG1RF, T13, 12107, and T20. The locally collinear blocks (LCBs) represent highly homologous regions and are shown with identical *colors*. The genomes were drawn to scale based on the genome of strain CBA7120

**Fig. 4:**
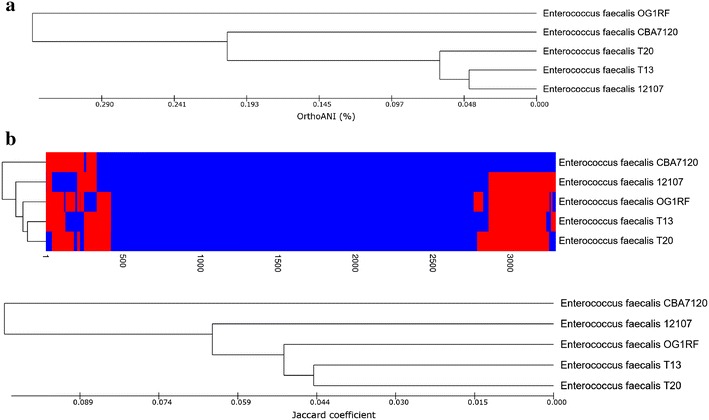
Dendrogram based on OrthoANI analysis and the presence of POGs in the genome of *Enterococcus faecalis* CBA7120 and the genomes of strains OG1RF, T13, 12107, and T20. **a** ANI phylogenetic tree. Using the orthologous average nucleotide identity tool, the phylogenetic tree was constructed based on OrthoANI values. **b** Dendrogram based on presence of POGs. Using Jaccard coefficients and UPGMA clustering, a dendrogram was generated. *Blue* indicates present genes and *red* indicates absent genes

**Fig. 5:**
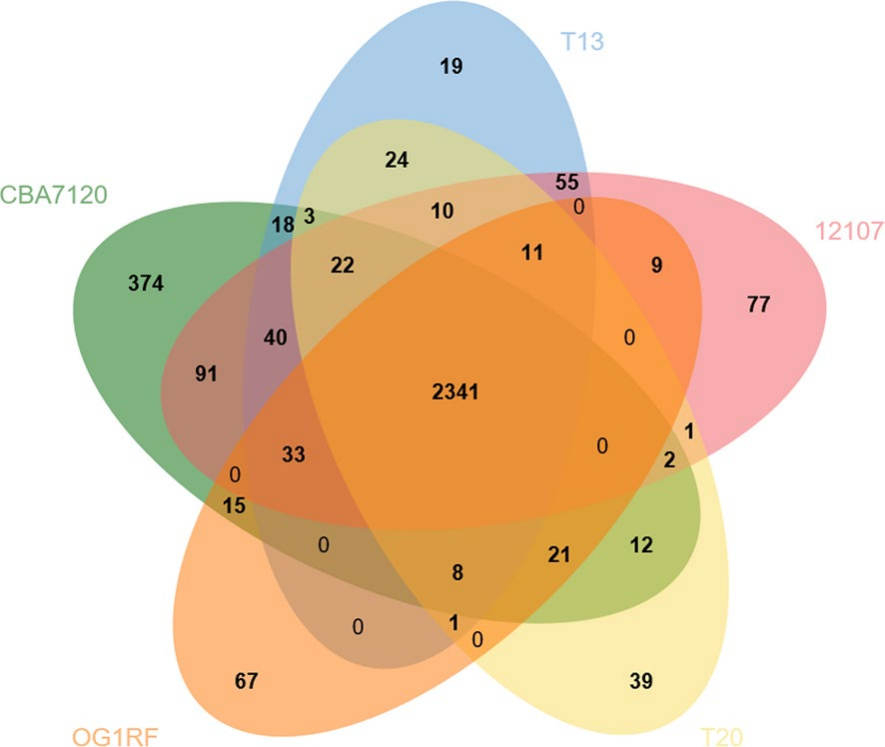
Venn diagram representing the pan-genomic landscape of *Enterococcus faecalis* CBA7120 and strains OG1RF, T13, 12107, and T20. The *numbers* in the Venn diagram indicate the number of POGs found to be shared among the indicated genomes

Whole-genome comparison of strain CBA7120 with *E. faecalis* 12107, T13, OG1RF, and T20 showed that most of the locally collinear blocks (LCBs) are highly homologous between the five assemblies, although the genome of strain CBA7120 contains large gaps in three LCBs (Fig. 3). *E. faecalis* CBA7120 showed 99.24, 99.55, 99.62 and 99.64% orthoANI values with *E. faecalis* strains OG1RF, T13, 12107, and T20, respectively. The phylogenetic tree based on orthoANI values for strain CBA7120 and the four reference strains indicated that strain CBA7120 is closely related to the *E. faecalis* reference strains (Fig. 4a). Based on analysis of the gene presence or absence heat map, strain CBA7120 possesses different POGs from the other four strains. In the dendrogram based on presence of POGs, strain CBA7120 was located as an outgroup to the other *E. faecalis* strains (Fig. 4b). As shown in Fig. 5, strain CBA7120 and the other four strains share 2341 POGs. The genome of strain CBA7120 contains only 374 POGs as singletons, contained in a single genome. Without the POGs that had no match in the SEED database, most of the 91 POGs identified belong to the “Phages, Prophages, Transposable elements, Plasmids” (20 POGs), “Carbohydrates” (12 POGs), “DNA metabolism” (11 POGs), and “Virulence, Disease and Defense” subsystems (9 POGs).

### Virulence factors

Based on comparison with the SEED database, 28 CDS were annotated as belonging to the “Virulence, Disease, Defense” category (Table 2). Strain CBA7120 was found to contain genes related to multidrug resistance efflux pumps, such as multidrug resistance efflux pump PmrA, multi antimicrobial extrusion protein, multidrug and toxin extrusion (MATE) family efflux pump YdhE/NorM, and multidrug-efflux transporter.

**Table 2:** Summary of protein coding sequences annotated as belonging to the “Virulence, Disease, and Defense” subsystem in the SEED database

Subsystem	Role	Location
*Streptococcus pyogenes* recombinatorial zone	Chaperonin (heat shock protein 33)	Contig 3
Colicin V and bacteriocin production cluster	Dihydrofolate synthase	Contig 3
	Folylpolyglutamate synthase	Contig 3
	Amidophosphoribosyltransferase	Contig 3
	Acetyl-coenzyme A carboxyl transferase beta chain	Contig 3
	Colicin V production protein	Contig 3
	tRNA pseudouridine synthase A	Contig 3
Copper homeostasis	Negative transcriptional regulator-copper transport operon	Contig 3
	Copper-translocating P-type ATPase	Contig 3
	Copper chaperone	Contig 3
Cobalt-zinc-cadmium resistance	DNA-binding heavy metal response regulator	Contig 3
	Cobalt-zinc-cadmium resistance protein	Contig 3
	Transcriptional regulator, MerR family	Contig 3
Resistance to fluoroquinolones	DNA gyrase subunit B	Contig 3
	DNA gyrase subunit A	Contig 3
	Topoisomerase IV subunit B	Contig 3
	Topoisomerase IV subunit A	Contig 3
Copper homeostasis: copper tolerance	Cytoplasmic copper homeostasis protein CutC	Contig 3
	Magnesium and cobalt efflux protein CorC	Contig 3
Beta-lactamase	Beta-lactamase class C and other penicillin-binding proteins	Contig 3
	Beta-lactamase	Contig 3
	Metal-dependent hydrolases of the beta-lactamase superfamily I	Contig 3
Cadmium resistance	Cadmium resistance protein	Contig 1
	Cadmium efflux system accessory protein	Contig 1
Multidrug resistance efflux pumps	Multidrug resistance efflux pump PmrA	Contig 3
	Multi antimicrobial extrusion protein [Na(+)/drug antiporter], MATE family of MDR efflux pumps	Contig 3
	Multidrug and toxin extrusion (MATE) family efflux pump YdhE/NorM, homolog	Contig 3
	Multidrug-efflux transporter, major facilitator superfamily (MFS)	Contig 3

### Future directions

Genomic analysis based on ortholog analysis may be a powerful comparative genomics tool. Additional study of the virulence factors present in *E. faecalis* CBA7120 will guide further research on *E. faecalis* virulence.

## Abbreviations

CDSs: coding DNA sequences
COG: clusters of orthologous groups
EG: Eggerth–Gagnon medium
LCB: locally collinear block
MATE: multidrug and toxin extrusion
MLST: multilocus sequence typing
POG: pan-genome orthologous group
RAST: rapid annotation using subsystem technology
RBH: reciprocal best hit
